# Technical note: TROG 15.01 SPARK trial multi‐institutional imaging dose measurement

**DOI:** 10.1002/acm2.12151

**Published:** 2017-08-02

**Authors:** Kimberley Legge, Peter B. Greer, Paul J. Keall, Jeremy T. Booth, Sankar Arumugam, Trevor Moodie, Doan T. Nguyen, Jarad Martin, Daryl John O'Connor, Joerg Lehmann

**Affiliations:** ^1^ School of Mathematical and Physical Sciences University of Newcastle Callaghan New South Wales Australia; ^2^ Radiation Oncology Department Calvary Mater Newcastle Waratah New South Wales Australia; ^3^ Radiation Physics Laboratory Sydney Medical School University of Sydney Camperdown New South Wales Australia; ^4^ Northern Sydney Cancer Centre Royal North Shore Hospital St Leonards New South Wales Australia; ^5^ Liverpool Cancer Therapy Centre Liverpool New South Wales Australia; ^6^ Crown Princess Mary Cancer Centre Westmead New South Wales Australia

**Keywords:** dose intercomparison, dose measurement, intrafraction motion monitoring, kilovoltage imaging dose

## Abstract

**Purpose:**

The Trans‐Tasman Radiation Oncology Group (TROG) 15.01 Stereotactic Prostate Adaptive Radiotherapy utilizing Kilovoltage intrafraction monitoring (SPARK) trial is a multicenter trial using Kilovoltage Intrafraction Monitoring (KIM) to monitor prostate position during the delivery of prostate radiation therapy. KIM increases the accuracy of prostate radiation therapy treatments and allows for hypofractionation. However, an additional imaging dose is delivered to the patient. A standardized procedure to determine the imaging dose per frame delivered using KIM was developed and applied at four radiation therapy centers on three different types of linear accelerator.

**Methods:**

Dose per frame for kilovoltage imaging in fluoroscopy mode was measured in air at isocenter using an ion chamber. Beam quality and dose were determined for a Varian Clinac iX linear accelerator, a Varian Trilogy, four Varian Truebeams and one Elekta Synergy at four different radiation therapy centers. The imaging parameters used on the Varian machines were 125 kV, 80 mA, and 13 ms. The Elekta machine was measured at 120 kV, 80 mA, and 12 ms. Absorbed doses to the skin and the prostate for a typical SBRT prostate treatment length were estimated according to the IPEMB protocol.

**Results:**

The average dose per kV frame to the skin was 0.24 ± 0.03 mGy. The average estimated absorbed dose to the prostate for all five treatment fractions across all machines measured was 39.9 ± 2.6 mGy for 1 Hz imaging, 199.7 ± 13.2 mGy for 5 Hz imaging and 439.3 ± 29.0 mGy for 11 Hz imaging.

**Conclusions:**

All machines measured agreed to within 20%. Additional dose to the prostate from using KIM is at most 1.3% of the prescribed dose of 36.25 Gy in five fractions delivered during the trial.

## 1 INTRODUCTION

Intrafraction motion during prostate radiation therapy treatment can reduce the dose to the prostate and increase the dose to organs at risk. Prostate position during treatment can be monitored using a variety of methods, including megavoltage (MV) imaging,^1^ ultrasound,^2^ Calypso electromagnetic guidance,^3^ the BrainLAB ExacTrac x‐ray system,^4^ the Cyberknife platform,^5^, ^6^, ^7^ and Navotek radioactive fiducials.^8^ Adjustments to either the patient or the beam position can then be made during treatment to correct for the motion observed.

Many of the methods available for prostate position monitoring require expensive equipment and expertise. Kilovoltage Intrafraction Monitoring (KIM) uses the gantry mounted kV imager available on modern linear accelerators and software installed on a framegrabber computer. KIM determines the position of the prostate in three dimensions from 2D kV projections using a probability density function.^9^, ^10^ KIM has successfully been used to measure prostate displacement during treatment in retrospective^11^ and interventional studies.^12^

The TROG 15.01 Stereotactic Prostate Adaptive Radiotherapy utilizing Kilovoltage intrafraction monitoring (SPARK) trial (https://clinicaltrials.gov/show/NCT02397317) is a multicenter trial using KIM to monitor prostate position during the delivery of hypofractionated, stereotactic prostate radiation therapy prescribed at 36.25 Gy to 95% of the planning target volume (PTV) delivered in five fractions. Four radiation therapy centers are participating in the trial, and are delivering treatments using Varian Clinac iX, Varian Trilogy, Varian Truebeam and Elekta Synergy linear accelerators. The imaging dose delivered during the use of KIM has previously been quantified using an IPEMB kV dose calculation formalism with CIRS phantom measurements^13^ and Monte Carlo simulations for a linear accelerator at a single center.^13^, ^14^

This work presents a simple method to assess the KIM kV imaging dose, which could be easily performed at multiple centers. The method uses standard, widely available equipment and an adaptation of the IPEMB protocol^15^ for kV dose measurement in air. Imaging dose delivered during the use of KIM by the different linear accelerator models used in the SPARK trial was then measured and assessed at all centers participating in SPARK.

## 2 METHOD

The method used for measurement of the kV imaging dose is based on the IPEMB protocol for kV dose measurements in air.^15^ This method was chosen as dose measurements needed to be acquired at four geographically separated centers with differing equipment available. The only required equipment is a 0.6 cc ionization chamber and holder with an electrometer, along with aluminum sheets for half‐value layer (HVL) measurements.

### 2.A Ion chamber measurements

Measurements were taken on seven different linacs: four Varian TrueBeams (Varian Medical Systems, Palo Alto, CA, USA), one Varian Clinac iX, one Varian Trilogy and one Elekta Synergy (Elekta AB, Stockholm, Sweden) at the four centers participating in the SPARK trial, all located in New South Wales, Australia. Measurements were acquired in air and used to calculate the imaging dose delivered to the prostate during prostate SBRT treatments delivered under the SPARK protocol.

For Varian linacs, dose measurements were taken using fluoroscopy mode at 125 kV, 80 mA, and 13 ms per frame. Measurements on the Elekta machine were taken using continuous acquisition mode at 120 kV, 80 mA, and 12 ms per frame.

**Figure 1:**
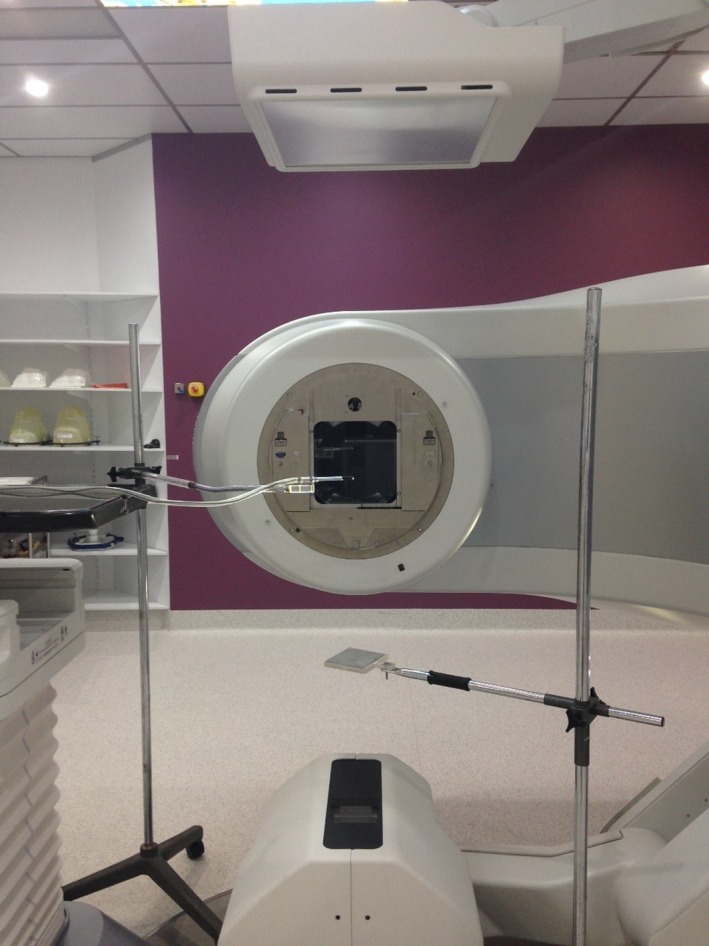
Experimental set up for ionization chamber measurements showing position of ionization chamber, kV source, and kV detector.

The beam quality was measured by finding the half value layer (HVL) of aluminum using a narrow 2 × 2 cm^2^ square field to reduce scatter. A farmer type ion chamber (without buildup cap) was placed at the isocenter 100 cm from the source and aligned to the center of the field using kV projections. The ion chamber remained in the same position for all HVL measurements. The attenuating material was placed 50 cm from the source. Care was taken to avoid scatter into the chamber by retracting the couch as far as possible and by extending the kV detector panel away from the ionization chamber. The equipment setup used for this measurement is shown in Fig. 1.

Dose was then measured at isocenter in air using the same setup (minus the holder for the attenuating material) and exposure settings for a field size of 6 × 6 cm^2^ (8 × 8 cm^2^ for the Synergy). The field size was chosen as it represents the field size used for KIM. Images were taken for 30 s in fluoroscopy mode at 11 fps. The number of frames measured was estimated from the time over which readings were taken multiplied by the number of frames acquired per second. The average ion chamber reading per frame was calculated from three readings.

### 2.B Calculation of dose in patient

The dose to water at the surface was calculated using eq. (1):

(1) $$D_{w,z=0,\mathrm{iso}}=MN_{k}\left(\frac{{\bar{\mu }}_{en}}{\rho }\right)B_{w}$$

where $D_{w,z=0}$ is the dose to water at surface, M is the ion chamber reading per frame corrected for temperature and pressure, $N_{k}$ is the chamber air kerma calibration factor, $\left(\frac{{\bar{\mu }}_{en}}{\rho }\right)$ is the mass energy absorption coefficient ratio (water to air) interpolated from the IPEMB protocol using the measured HVL and $B_{w}$ is the back scatter factor, also from the IPEMB protocol.^15^

Crocker et al.^13^ found that for a cohort of 22 patients set up for treatment with the center of their prostate at isocenter, the median source to surface distance (SSD) was 84.7 cm and the median PTV depth was at 15.3 cm below the surface. Percentage depth dose (PDD) at 100 cm SSD within a CIRS phantom setup for a 10 × 10 cm^2^ field at 15 cm depth was measured in that study to be 10%.^13^

Therefore, an inverse square law (ISL) factor was first applied to determine the dose to the water at surface at an SSD of 84.7 cm from the measured dose to water surface at isocenter (100 cm). Then the percentage depth dose was used to determine the dose at depth 15.3 cm at isocenter within the phantom (eq. (2)):

(2) $$D_{w,z=15,iso}=D_{w,z=0,iso}.ISL.PDD.FSF$$

Where $D_{w,z=0,iso}$ is the dose to surface at isocenter found using equation (1) above, ISL is the inverse square law factor, PDD is the percentage depth dose factor and FSF is the field size factor. This method makes the assumption that the dose delivered to the isocenter is uniform throughout the whole prostate and that the prostate is of uniform depth in the patient at all gantry (kV source) angles during VMAT treatment.

The dose delivered per treatment, $D_{\mathrm{iso},\;\mathrm{treatment}},$ was calculated using eq. (3):

(3) $$D_{\mathrm{iso},\;\mathrm{treatment}}=D_{w,z=15,iso}.fps.t_{treatment}$$

where fps is the number of kV frames acquired per second (images can be acquired at 1, 5, or 11 Hz) and $t_{treatment}$ is the estimated treatment length. Treatment length for each 7.25 Gy fraction delivered with VMAT at a maximum dose rate of 600 MU/min was determined by measurement of beam on time during the delivery of test plans created according to the SPARK protocol. The average beam on time was 314 s.

### 2.B Estimation of skin dose

The dose delivered to each section of skin was estimated by assuming a cylindrical patient geometry with a radius of 15.3 cm. The dose per frame to skin was determined using eq. (4):

(4) $$D_{w,z=0}=D_{w,z=0,iso}.ISL$$

where $D_{w,z=0,iso}$ is the dose delivered to the surface at isocenter calculated using eq. (1) and ISL is the inverse square law factor used to scale the dose at isocenter to the dose delivered at a point 15.3 cm above isocenter (1.39).

Taking into account beam divergence and assuming fractions are delivered using two partial arcs, each of 280°, the skin dose delivered to a section of skin by a 6 × 6 cm^2^ kV beam can be estimated using Equation (5):

(5) $$D_{skin}=t_{exposed}.fps.D_{w,z=0}$$

where $t_{exposed}$ is the time in seconds for which each point of skin is exposed to the kV beam, fps is the number of kV frames acquired per second and $D_{w,z=0}$ is the dose per frame delivered to the skin surface calculated using eq. (4).

The value for $t_{exposed}$ can be found using the beam on time and the width of the beam at the skin surface. The width is determined using trigonometry as shown in eq. (6):

(6) $$w=SSD\left(\frac{x}{100}\right)$$

where w is the width of the beam at the skin surface, SSD is the source to surface distance and x is the field size.

The time that each patch of skin is exposed to the beam was then determined using eq. (7):

(7) $$t_{exposed}=\frac{w.360}{c}.\frac{t_{total}}{angle}$$

where $t_{exposed}$ is the time that each patch of skin is exposed to the beam, w is the width of the beam at the skin surface, c is the patient circumference, $t_{total}$ is the time taken to deliver all arcs making up the entire treatment and angle is the angle subtended by each individual arc delivered. In this case, for a field size of 6 × 6 cm^2^, SSD of 83.7 cm, treatment time of 314 s, arc angle of 280° and assuming a patient radius of 15.3 cm, the resulting $t_{exposed}$ was found to be 21.4 s.

## 3 RESULTS

The half value layer (HVL), dose per frame at isocenter at 15.3 cm depth and absorbed dose to the prostate at different imaging frequencies delivered by each of the seven linear accelerators measured using an ion chamber are listed in Table 1.

**Table 1:** HVL and per‐frame and total treatment course (five fractions) dose values for each Linac obtained using ion chamber measurements. Calculated total dose assumes a 314 s treatment time for each of five fractions delivered. TB refers to Varian Truebeam machines

Vendor	Linac	kV imaging settings	HVL (mm Al)	D_w,z=0, iso_ (mGy/frame)	D_w,z=15, iso_ (mGy/frame)	D_total_ (1 Hz) (mGy)	D_total_ (5 Hz) (mGy)	D_total_ (11 Hz) (mGy)
Varian	Clinac iX	125 kV, 1.04 mAs per frame	4.95	0.16	0.023	35.9	179.4	394.7
	Trilogy		4.7	0.17	0.023	36.7	183.7	404.2
	TB 1		4.4	0.19	0.027	41.9	209.4	460.6
	TB 2		4.65	0.20	0.028	43.7	218.6	480.9
	TB 3		4.68	0.19	0.026	41.0	205.0	451.0
	TB 4		4.7	0.19	0.026	41.4	206.9	455.2
Elekta	Synergy	120 kV, 0.96 mAs per frame	7.5	0.09	0.025	38.9	194.7	428.3

The estimated dose delivered to a point on the skin by each machine at each frame rate appears in Table 2. The results are for the whole course of treatment (five treatment fractions).

**Table 2:** Estimated dose delivered to a point on the skin

Vendor	Linac	D_Skin_ (1 Hz) (mGy)	D_Skin_ (5 Hz) (mGy)	D_Skin_ (11 Hz) (mGy)
Varian	Clinac iX	24.5	122.3	269.0
	Trilogy	25.0	125.2	275.5
	TB 1	28.5	142.7	313.9
	TB 2	29.8	149.0	327.8
	TB 3	27.9	139.7	307.4
	TB 4	28.2	141.0	310.3
Elekta	Synergy	27.6	130.2	304.1

The uncertainty of the measurements was estimated based on the limit of reading of the electrometers used, uncertainty in thickness of materials used for HVL measurements, and uncertainties resulting from estimation of the number of frames measured. The uncertainty in the measured doses is approximately 7%.

## 4 DISCUSSION

Imaging dose delivered during the use of kilovoltage intrafraction monitoring for prostate SBRT treatments was measured at four different radiation therapy centers and on seven linear accelerators from two vendors. Limited comparisons between imaging dose delivered by different machines or at different centers exist in the literature, and such reports focus on the dose given for CBCT scans.^16^, ^17^

The method presented here allows for simple, quick measurement of kV imaging dose at geographically separated centers. The standardized procedure used minimal equipment, namely a 0.6 cc ionization chamber, a chamber holder, an electrometer and aluminum sheets, all of which is readily available at most radiation therapy centers.

Doses measured for linacs of the same type (Varian Clinac and Varian TrueBeam) agreed within the measurement uncertainty. The absorbed kV dose delivered by all machines from all vendors agreed to within 18%, with Varian Truebeam and Elekta Synergy machines delivering a higher dose than the Varian Clinac iX and Varian Trilogy. The largest imaging dose measured, with an imaging rate of 11 Hz, was 92.1 mGy per fraction (314 s) to the prostate, giving a total dose of 460.7 mGy over the entire course of stereotactic prostate treatment (five fractions). This is approximately 1.3% of the prescribed dose to the prostate in the case of the 36.25 Gy used in the SPARK trial. The average imaging dose delivered across all machines using a frame rate of 11 Hz was 87.9 ± 5.8 mGy per fraction. Although the use of KIM requires an additional imaging dose to be delivered to patients, this dose provides dosimetric advantages in terms of target dose coverage and reduced dose to organs at risk which are gained by gating the treatment beam to adjust for target motion.^18^

The imaging dose delivered during the use of this localization method can be reduced significantly by reducing the imaging frequency. Imaging at 1 Hz has been shown to provide sufficient localization accuracy for prostate treatments when using KIM.^10^ Lowering the imaging frequency requires methods to reduce the level of MV scatter on the kV detector. This can be achieved by a dark frame readout immediately prior to kV image acquisition.^19^ Reducing the kV field size based on individual patient seed placement following the method outlined by Crocker et al. can also provide reductions in imaging dose.^13^ Further reductions in imaging dose could be obtained using both patient‐specific and gantry angle‐specific kV and mAs settings. For example, the task of finding fiducial markers in an anterior**–**posterior kV image where the anatomical pathlength is short and the absence of bony anatomy within the beam requires much lower dose than achieving the same task for lateral beams.

The reduction in treatment time achieved by hypofractionating treatments and using a VMAT technique provides a significant reduction in imaging dose delivered while using KIM. The imaging dose determined for 1 Hz, 120 kV, 1.04 mAs imaging during KIM for IMRT prostate treatments has previously been reported as 185 mGy across the whole course of treatment.^13^ For hypofractionated prostate SBRT treatments under the SPARK protocol, the dose for 1 Hz kV imaging at 125 kV 1.04 mAs during the use of KIM is a maximum of 44 mGy across the whole treatment course (five fractions, 314 s per fraction) for the linear accelerators measured in this study. This is a reduction in imaging dose of 76% achieved by changing to a hypofractionated VMAT treatment technique. Treatment time, and hence imaging dose resulting from the use of KIM, could be further reduced by the use of higher dose rates achievable with flattening filter free beams where they are available.

The absorbed dose to the prostate during a pelvic CBCT scan has been measured to be 27.63 mGy on the Elekta XVI system and 27.25 mGy on the Varian OBI system.^16^ If one CBCT is performed per treatment fraction, patients treated on an Elekta machine would receive a CBCT dose of 138.2 mGy to the prostate over a five fraction course of treatment, while patients treated using a Varian linac would receive a CBCT dose of 136.3 mGy. This is significantly larger than the dose delivered using KIM at 1 Hz.

The skin dose delivered using KIM was estimated to be a maximum of 327.8 mGy to each point on the skin over the entire course of treatment when KIM imaging occurs at 11 Hz. Penoncello and Ding (2016) determined the average patient skin dose delivered by the MV treatment beam for a variety of prostate VMAT plans to be 8616.7 ± 1092.8 mGy,^20^ far above the estimated skin dose delivered as a result of KIM imaging. Deterministic skin effects may occur for skin doses of 2 Gy and above. The maximum dose likely to be delivered as a result of KIM imaging falls far below this number.

The Cyberknife system uses two kilovoltage imagers to track the anatomical or fiducial movements in real time so that the linac direction can be adjusted to account for organ motion. In one Cyberknife fraction, between 30 and 50 image pairs are normally acquired, and the entrance surface dose per projection is between 0.25 and 2 mGy, depending on the treatment site and imaging method used.^21^ The maximum entrance dose per projection measured for KIM in this study is slightly lower than the lowest Cyberknife value, however, at least three times as many images are likely to be taken when using KIM during a VMAT treatment, and so the overall surface dose is likely to be on the same order of magnitude. It should be noted, however, that the skin dose during a KIM VMAT treatment will be spread around the patient as the gantry moves, while the Cyberknife is likely to deliver a more concentrated dose to just some regions of the skin.

## 5 CONCLUSIONS

Dose delivered to the prostate due to KIM was measured and quantified for seven different linear accelerators at four radiation therapy centers using a simple in‐air measurement method based on the IPEMB protocol. The imaging dose delivered by all machines at all centers agreed to be within 20%. The average absorbed dose to the prostate for the whole five fraction SBRT treatment across all machines measured was 39.9 ± 2.6 mGy for 1 Hz imaging, 199.7 ± 13.2 mGy for 5 Hz imaging, and 439.3 ± 29.0 mGy for 11 Hz imaging.

## CONFLICTS OF INTEREST

Royal North Shore Hospital discloses collaborative research agreement with Varian Medical Systems providing in‐kind loan equipment for this study.

Paul Keall is an inventor on an issued patent related to the KIM technology that is licensed from Stanford University to Varian Medical Systems. He is an author on a second issued patent related to the KIM technology that is unlicensed. Paul Keall is supported by an NHMRC Fellowship. The SPARK trial is supported by Cancer Australia.

Kimberley Legge is the recipient of an Australian Postgraduate Award.

## References

[1] Berbeco RI , Hacker F , Ionascu D , Mamon HJ . Clinical feasibility of using an EPID in cine mode for image‐guided verification of stereotactic body radiotherapy. Int J Radiat Oncol Biol Phys. 2007;69:258–266. 17707280 10.1016/j.ijrobp.2007.04.051

[2] Schlosser J , Salisbury K , Hristov D . Telerobotic system concept for real‐time soft‐tissue imaging during radiotherapy beam delivery. Med Phys. 2010;37:6357–6367. 21302793 10.1118/1.3515457

[3] Kupelian P , Willoughby T , Mahadevan A , et al. Multi‐institutional clinical experience with the Calypso System in localization and continuous, real‐time monitoring of the prostate gland during external radiotherapy. Int J Radiat Oncol Biol Phys. 2007;67:1088–1098. 17187940 10.1016/j.ijrobp.2006.10.026

[4] Jin J‐Y , Yin F‐F , Tenn SE , Medin PM , Solberg TD . Use of the BrainLAB ExacTrac x‐ray 6D system in image‐guided radiotherapy. Med Dosim. 2008;33:124–134. 18456164 10.1016/j.meddos.2008.02.005

[5] Oermann EK , Slack RS , Hanscom HN , et al. A pilot study of intensity modulated radiation therapy with hypofractionated stereotactic body radiation therapy (SBRT) boost in the treatment of intermediate‐ to high‐risk prostate cancer. Technol Cancer Res Treat. 2010;9:453–462. 20815416 10.1177/153303461000900503

[6] Katz AJ , Santoro M , Ashley R , Diblasio F , Witten M . Stereotactic body radiotherapy as boost for organ‐confined prostate cancer. Technol Cancer Res Treat. 2010;9:575–582. 21070079 10.1177/153303461000900605

[7] Jabbari S , Weinberg VK , Kaprealian T , et al. Stereotactic body radiotherapy as monotherapy or post–external beam radiotherapy boost for prostate cancer: technique, early toxicity, and PSA response. Int J Radiat Oncol Biol Phys. 2012;82:228–234. 21183287 10.1016/j.ijrobp.2010.10.026

[8] Shchory T , Schifter D , Lichtman R , Neustadter D , Corn BW . Tracking accuracy of a real‐Time fiducial tracking system for patient positioning and monitoring in radiation therapy. Int J Radiat Oncol Biol Phys. 2010;78:1227–1234. 20615628 10.1016/j.ijrobp.2010.01.067

[9] Poulsen PR , Cho B , Keall PJ . A method to estimate mean position, motion magnitude, motion correlation, and trajectory of a tumor from cone‐beam CT projections for image‐guided radiotherapy. Int J Radiat Oncol Biol Phys. 2008;72:1587. 19028282 10.1016/j.ijrobp.2008.07.037

[10] Poulsen PR , Cho B , Keall PJ . Real‐time prostate trajectory estimation with a single imager in arc radiotherapy: a simulation study. Phys Med Biol. 2009;54:4019. 19502704 10.1088/0031-9155/54/13/005

[11] Ng JA , Booth JT , Poulsen PR , et al. Kilovoltage intrafraction monitoring for prostate intensity modulated arc therapy: first clinical results. Int J Radiat Oncol Biol Phys. 2012;84:e655–e661. 22975613 10.1016/j.ijrobp.2012.07.2367 PMC5357433

[12] Keall PJ , Aun Ng J , O'Brien R , et al. The first clinical treatment with kilovoltage intrafraction monitoring (KIM): a real‐time image guidance method. Med Phys. 2015;42:354–358. 25563275 10.1118/1.4904023

[13] Crocker JK , Ng JA , Keall PJ , Booth JT . Measurement of patient imaging dose for real‐time kilovoltage x‐ray intrafraction tumour position monitoring in prostate patients. Phys Med Biol. 2012;57:2969–2980. 22517054 10.1088/0031-9155/57/10/2969 PMC3369877

[14] Ng JA , Booth J , Poulsen P , Kuncic Z , Keall PJ . Estimation of effective imaging dose for kilovoltage intratreatment monitoring of the prostate position during cancer radiotherapy. Phys Med Biol. 2013;58:5983–5996. 23938470 10.1088/0031-9155/58/17/5983 PMC5357434

[15] Klevenhagen SC , Aukett RJ , Harrison RM , Moretti C , Nahum AE , Rosser KE . The IPEMB code of practice for the determination of absorbed dose for x‐rays below 300 kV generating potential (0.035 mm Al–4 mm Cu HVL; 10–300 kV generating potential). Phys Med Biol. 1996;41:2605–2625. 15930599 10.1088/0031-9155/50/12/001

[16] Hyer DE , Serago CF , Kim S , Li JG , Hintenlang DE . An organ and effective dose study of XVI and OBI cone‐beam CT systems. J Appl Clin Med Phys. 2010;11:3183. 20592702 10.1120/jacmp.v11i2.3183 PMC5719945

[17] Song WY , Kamath S , Ozawa S , et al. A dose comparison study between XVI^®^ and OBI^®^ CBCT systems. Med Phys. 2008;35:480–486. 18383668 10.1118/1.2825619

[18] Colvill E , Poulsen PR , Booth JT , O'Brien RT , Ng JA , Keall PJ . DMLC tracking and gating can improve dose coverage for prostate VMAT. Med Phys. 2014;41:091705. 25186380 10.1118/1.4892605

[19] Poulsen PR , Jonassen J , Schmidt ML , Jensen C . Improved quality of intrafraction kilovoltage images by triggered readout of unexposed frames. Med Phys. 2015;42:6549–6557. 26520745 10.1118/1.4933248

[20] Penoncello GP , Ding GX . Skin dose differences between intensity‐modulated radiation therapy and volumetric‐modulated arc therapy and between boost and integrated treatment regimens for treating head and neck and other cancer sites in patients. Med Dosim. 2016;41:80–86. 26764180 10.1016/j.meddos.2015.09.001

[21] Murphy MJ , Balter J , Balter S , et al. The management of imaging dose during image‐guided radiotherapy: report of the AAPM task group 75. Med Phys. 2007;34:4041–4063. 17985650 10.1118/1.2775667

